# Assessing antibiotic sorption in soil: a literature review and new case studies on sulfonamides and macrolides

**DOI:** 10.1186/1752-153X-8-5

**Published:** 2014-01-17

**Authors:** Stacia R Wegst-Uhrich, Divina AG Navarro, Lisa Zimmerman, Diana S Aga

**Affiliations:** 1 Department of Chemistry, University at Buffalo, State University of New York, Buffalo, NY 14260, USA; 2 Commonwealth Scientific and Industrial Research Organization, Private Bag 2, Glen Osmond, SA 5064, Australia

**Keywords:** Veterinary pharmaceuticals, Antibiotics, Antimicrobials, Sulfamethazine, Tylosin, Partition coefficient, Sorption, Mobility, Degradation, Transformation

## Abstract

The increased use of veterinary antibiotics in modern agriculture for therapeutic uses and growth promotion has raised concern regarding the environmental impacts of antibiotic residues in soil and water. The mobility and transport of antibiotics in the environment depends on their sorption behavior, which is typically predicted by extrapolating from an experimentally determined soil-water distribution coefficient (K_d_). Accurate determination of K_d_ values is important in order to better predict the environmental fate of antibiotics. In this paper, we examine different analytical approaches in assessing K_d_ of two major classes of veterinary antibiotics (sulfonamides and macrolides) and compare the existing literature data with experimental data obtained in our laboratory. While environmental parameters such as soil pH and organic matter content are the most significant factors that affect the sorption of antibiotics in soil, it is important to consider the concentrations used, the analytical method employed, and the transformations that can occur when determining K_d_ values. Application of solid phase extraction and liquid chromatography/mass spectrometry can facilitate accurate determination of K_d_ at environmentally relevant concentrations. Because the bioavailability of antibiotics in soil depends on their sorption behavior, it is important to examine current practices in assessing their mobility in soil.

## Introduction

Veterinary pharmaceuticals (VPs) are physiologically active compounds that are used to protect animals against parasites, prevent bacterial infections, and growth promotion [1-6]. Antibiotics, their synthetic analogues, and synthetically produced antimicrobials are introduced in both therapeutic and medical dosages to the animals through medicated feeds, injections, and external application [1]. Most animals do not absorb these compounds completely; hence antibiotics are excreted in urine and feces as parent compounds, and in mixtures with their conjugated metabolites and oxidation/hydrolysis products [1,3-8].

The presence of antibiotic residues in animal manure that are land-applied to agricultural lands can contaminate water and soil [9-11]. Consequently, changes in the soil microbial population can occur; the microorganisms’ ability to degrade contaminants and their role in chemical cycles, such as nitrification, may be affected significantly [5,7,12-14]. Persistent antibiotics can accumulate in the top layers of soil, may leach to the groundwater, or can be transported to surface waters [1,15,16]. Sulfonamide antibiotics, the first broad spectrum antibacterial medications [17], are relatively persistent in the environment and do not sorb strongly to soil. Thus, sulfonamides have been detected in surface water, ground water, soil pore water [18-20], and drinking water [21] that have been impacted by agricultural and human activities.

A common parameter used to predict the transport behavior of organic contaminants in soil is the soil-water partition coefficient, K_d_. The K_d_ values can be directly determined experimentally, or derived indirectly from the octanol-water partition coefficients (K_ow_) or by computational modeling using free energy calculations. Because the sorption properties of antibiotics affect their mobility and ecotoxicology, it is important to recognize that different K_d_ measurements may provide varying results that could potentially lead to large errors in environmental models that are used in risk assessment.

The fate and transport of antibiotics in the environment depend on the underlying physical properties of the compound such as water solubility, lipophilicity, volatility, and sorption potential. Soil can act as potential sink, and thus sorption of antibiotics in the solid phase can reduce their mobility, reactivity, and bioavailability for microbial degradation [22]. In addition, soil properties such as organic carbon content, ionic strength, clay content, texture, and pH can alter sorption mechanisms involved, and the extent of sorption of antibiotics [23]. The assumption that sorption occurs solely through hydrophobic partitioning to soil organic matter (OM) is inappropriate for antibiotics with ionizable groups, when electrostatic interactions and hydrogen bonding become significant [1].

The purpose of this paper is to provide a review of the different ways that K_d_ values are measured, and demonstrate how the analytical differences may affect the prediction of the fate and transport of antibiotics in the environment. Specifically, this review will focus on two of the most used classes of antibiotics: sulfonamides and macrolides. Within these classes, sulfamethazine, tylosin, and erythromycin are examined due to their wide use in animal related practices, and their variable sorption properties [1,24].

## Review

### Octanol-water partition coefficient (K_ow_) and octanol-water distribution ratio (D_ow_)

Antibiotic mobility in soil has been traditionally estimated using the octanol-water partition coefficient (K_ow_):

$$K_{\mathrm{ow}}=\frac{{\left[\mathrm{Solute}\right]}_{\mathrm{octanol}}}{{\left[\mathrm{Solute}\right]}_{\mathrm{water}}}$$

However, K_ow_ only reflects hydrophobic interactions and does not accurately account for electrostatic interactions, surface complexation, hydrogen bonding, cation-exchange, or bridging that may vary significantly with changes in pH, OM, and ionic strength [1]. While the use of K_ow_ in predicting soil sorption behavior of nonpolar compounds works fairly well, the application of K_ow_ for polar or ionizable compounds, such as many antibiotics, may be inaccurate. Additionally, the variety of environmental factors (such as soil properties) that can affect sorption will complicate the modeling efforts to predict sorption and mobility of antibiotics. For example, OM may block interlayer sites of clay minerals [25], but such phenomenon is not accounted for by K_ow_ values. Thus simply using K_ow_ will result in incorrect assessment of antibiotic fate and transport in the environment.

The pH dependent octanol-water distribution ratio D_ow,_ can be used to avoid variations in K_ow_ values resulting from changes in pH. The D_ow_ value considers hydrophobicity and ionogenicity, and is a combination of the K_ow_ (of the neutral compound) and the pK_a_, in which the transfer of both neutral and ionized species between the aqueous and immiscible phase is accounted for [26]. The D_ow_ value does not consider hydrophobicity as the sole governing factor that dictates partitioning of neutral compounds, but also accounts for the transfer of ion pairs and free ions from aqueous to the organic layer [27,28]:

$$D_{\mathrm{ow}}=\frac{{\left[\mathrm{nonionized}+\mathrm{ionized}\;\mathrm{species}\right]}_{\mathrm{octanol}}}{{\left[\mathrm{nonionized}+\mathrm{ionized}\;\mathrm{species}\right]}_{\mathrm{water}}}$$

A relationship between log K_ow_ and log D_ow_ can be derived for both acidic and basic compounds [29]. For sulfamethazine, K_ow_ values between 1.042 and 3.750 are reported, while D_ow_ values between 0.427 and 1.950 are reported (determined at pH values of 4-8). These K_ow_ and D_ow_ values were calculated using Advanced Chemistry Development (ACD/Labs) Software V11.02 (© 1994-2012 ACD/Labs). Notably, these values fall in the lower part of the wide range of K_d_ values (0.23-30 L/kg) obtained experimentally, as reported from literature (Table 1).

**Table 1:** Sorption coefficients of sulfamethazine

**Soil type**	**K**_**d**_**(L/kg)**	**Concentration range used**	**Measurement technique**	**Reference**
Sandy loam	0.27-0.77	1.5, 3.5, 7.5, 10, 15 ppm	HPLC-UV (λ = 275 nm)	[34]
	0.23-1.22	0.3 - 20 ppm	HPLC-UV (λ = 254 nm)	[33]
	9.8-22	0, 0.25, 0.50, 1.0, 2.5, 4.0 ppm	HPLC-UV (λ = 275 nm)	[32]
	0.95-19.53	17.7, 35.4, 53.1, 70.8, 88.5 ppb	Liquid scintillation counting	ED
			(LSC) with ^14^C-SMZ	Method 1
	1.0-7.52	1, 3, 10, 20, 30, 50, 100, 300 ppb	LSC with ^14^C-SMZ	ED
				Method 1
Clay loam	2.88	1.5, 3.5, 7.5, 10, 15 ppm	HPLC-UV (λ = 275 nm)	[34]
	16.55 ± 1.41	0.012, 0.122, 1.219 ppm	LSC with ^14^C-SMZ	[31]
Loam	1.05-3.91	0.3 - 20 ppm	HPLC-UV (λ = 254 nm)	[33]
	3.1-17	0, 0.25, 0.50, 1.0, 2.5, 4.0 ppm	HPLC-UV (λ = 275 nm)	[32]
	2.83-22.28	17.7, 35.4, 53.1, 70.8, 88.5 ppb	Liquid scintillation counting	ED
			(LSC) with ^14^C-SMZ	Method 1
	0.9-18.2	1, 3, 10, 20, 30,	LSC with ^14^C-SMZ	ED
		50, 100, 300 ppb		Method 1
	17.10 ± 1.66	0.012, 0.122, 1.219 ppm	LSC with ^14^C-SMZ	[31]
Silty clay loam	18.58 ± 2.29	0.012, 0.122, 1.219 ppm	LSC with ^14^C-SMZ	[31]
Silt loam	0.82-2.12	1.5, 3.5, 7.5, 10, 15 ppm	HPLC-UV (λ = 275 nm)	[34]
	0.66 - 6.73	2.5 – 50 μM	HPLC-UV (λ = 254 nm)	[30]
			LSC with ^14^C-SMZ	
	206.18 ± 12.09	0.012, 0.122, 1.219 ppm	LSC with ^14^C-SMZ	[31]
Loamy sand	2.3-30	0, 0.25, 0.50, 1.0, 2.5, 4.0 ppm	HPLC-UV (λ = 275 nm)	[32]
Sand	7.52 ± 0.09	0.012, 0.122, 1.219 ppm	LSC with ^14^C-SMZ	[31]

Note: Results are soil dependent. ED: experimentally determined. Methodology can be found in the additional files. K_ow_ of sulfamethazine ranges from 1.042-3.750. K_ow_ values calculated using Advanced Chemistry Development (ACD/Labs) Software V11.02 (© 1994-2012 ACD/Labs).

### The K_d_ partition coefficient

The soil-water partition coefficient (K_d_) is used to describe the sorption potential of pollutants and the extent that they will move into the ground or surface waters. Using K_d_ instead of K_ow_ demonstrates sorption behavior with respect to the soil media of interest, and data extrapolation from the octanol to soil matrices is eliminated. The K_d_ value is the ratio between the concentration of the compound in soil (C_s_) [total concentration, including sorbed transformation products] to the concentration of the dissolved compound in water (C_w_) [1]:

$$K_{d}=\frac{C_{s}}{C_{w}}$$

In the experimental determination of K_d_ values, it is important to accurately measure the concentrations of the compounds at environmentally relevant levels for both the water and soil components to assure mass balance. K_d_ is typically determined one of two ways: (1) column displacement studies in which determination occurs from a breakthrough curve at a single location, or (2) batch sorption experiments in which multiple concentrations are used to construct isotherms by plotting C_s_ versus C_w_.

**Figure 1:**
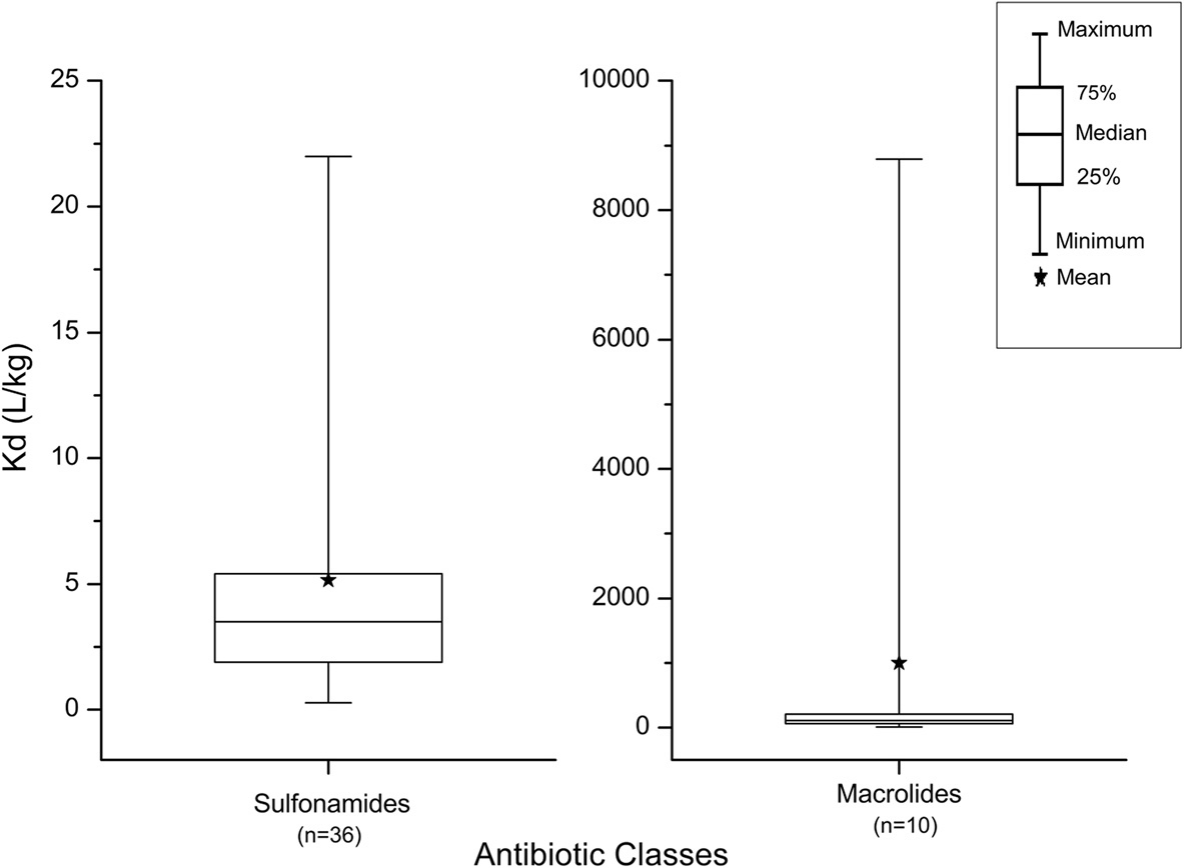
**Box plots of K**_**d **_**values for sulfonamides and macrolides reported in literature.** The sulfonamides (left) include sulfamethazine and sulfachloropyridazine, and the macrolides (right) includes tylosin and erythromycin. pH values range from 5.2-7.5 when reported. Soil types include loamy and sandy loam, clay loam, loam, loamy sand, and silt loam. The high variation of K_d_ values found in literature is illustrated here. The upper and lower boundaries of the box represent the 75^th^ and 25^th^ percentile respectively. The middle line indicates the median value, and the whiskers indicate the maximum and minimum values.

Experimentally determined K_d_ values reported in the literature for a particular compound are highly variable even for the same soil type and environmental conditions. For example, Tables 1 and 2 list K_d_ values for sulfamethazine, a sulfonamide [30-34] and tylosin, a macrolide [2,6,25,35-38], respectively. Corresponding plots were drawn in Figure 1 to clearly demonstrate the wide range of their K_d_ values reported. For the same type of soil, K_d_ values appear to vary widely depending on the concentration ranges used to determine K_d_.

**Table 2:** Sorption coefficients of tylosin

**Soil Type**	**K**_**d**_**(L/kg)**	**Concentration range used**	**Measurement technique**	**Reference**
Sandy Loam	58.1 – 148.0	500 ppb	LC-MS	[37]
			LC-fluorescence	
	92	1000 ppm	ELISA	[35]
	101.02-13961.00	10, 100, 200, 1000 ppb	HPLC-MS	ED
			(ion trap)	Method 2
	6737-33871	1, 5, 10, 100, 200, 500, 1000 ppb	HPLC-MS/MS	ED
				Method 3
	42	5 ppm	HPLC-UV	[36]
			(λ = 290 nm)	
Clay Loam	66	1000 ppm	ELISA	[35]
	156 ± 8	3 ppm	HPLC-MS (SIM)	[6]
	1.76 – 6.19	0.5, 5, 50 ppm	HPLC-UV	[25]
			(λ = 290 nm)	
	65	5 ppm	HPLC-UV	[36]
			(λ = 290 nm)	
Loam	59.35-1176.00	10, 100, 200, 1000 ppb	HPLC-MS (ion trap)	ED
				Method 2
	1684-172480	1, 5, 10, 100, 200, 500, 1000 ppb	HPLC-MS/MS	ED
				Method 3
	5.77 - 12.4	0.5, 5, 50 ppm	HPLC-UV	[25]
			(λ = 290 nm)	
Loamy Sand	8.3 ± 1.2	500 ppb	LC-MS	[37]
			LC-fluorescence	
	8.9 ± 0.4	3 ppm	HPLC-MS (SIM)	[6]
Sand Soil	10.8 ± 0.7	500 ppb	LC-MS	[37]
			LC-fluorescence	
	24	5 ppm	HPLC-UV	[36]
			(λ = 290 nm)	
Clay%: 0.2 - 51.6%	10.4-387.0	3 - 7.5 ppm	HPLC-MS (SIM)	[38]
	(Average = 129.5)			
Clay%: < 3 - 69% (K_d_ increases with clay %)	2.23-5520	5 - 43 μmol/L	HPLC-UV,	[2]
	(Tylosin A)		(λ = 280 nm)	
	547-4745	5 - 43 μmol/L	HPLC-UV,	[2]
	(Tylosin D)		(λ = 280 nm)	
	597-6520	5 - 43 μmol/L	HPLC-UV,	[2]
	(Tylosin A-Aldol)		(λ = 280 nm)	

Note: Results are soil dependent. ED: experimentally determined. Methodology can be found in the additional files. K_ow_ ranges for tylosin A and it’s metabolites: tylosin A: 0.552-32.6587; tylosin B: 1.535-78.343; tylosin C: 0.676-41.495;tylosin D: 0.309-17.947. K_ow_ values calculated using Advanced Chemistry Development (ACD/Labs) Software V11.02 (© 1994-2012 ACD/Labs).

In general, K_d_ values have been obtained using high concentrations (in the parts per million range) of antibiotics that are not environmentally relevant. High concentrations are used to enable detection of the desorbed portion without sample pre-concentration. However, the use of high concentrations of antibiotics for sorption experiments can result in anomalies when the K_d_ value is concentration-dependent and exhibits non-linearity. For example, the K_d_ value for sulfachloropyridazine (pk_a_ = 1.88, 5.90) was determined in sandy loam (pH 6.0-7.5 and 6.6, respectively) at 1-10 ppb to be 0.9 L/kg, while when determined at 1.5 ppm the K_d_ value was 8.1 L/kg. These discrepancies in the K_d_ values pose differences in predicting the fate of sulfachloropyridazine; it implies that at lower concentrations, sulfachloropyridazine is considered to have high mobility under the pesticide mobility classification [6,19], while at higher concentrations sulfachloropyridazine has low mobility [6,39].

### The freundlich sorption constant, K_f_

Because sorption coefficients are not always the same at all aqueous concentrations, linear plots are not always observed. In the case of tylosin, non-linearity has been previously reported [40,41], and therefore all original data will be presented as both K_d_ and K_f_ values. The Freundlich constant (sorption coefficient) K_f_ provides a better estimate of partitioning:

$$K_{f}={\frac{C_{s}}{C_{w}}}^{1-n}$$

where *n*, the Freundlich exponent, is a measure of the isotherm nonlinearity. A plot of log C_s_ vs. log C_w_ gives a linear isotherm with a slope equal to *n* and a y-intercept equal to log K_f_. If the value of K_f_ approaches the value of K_d_, the Freundlich exponent, *n*, is equal to 1, and sorption is linear. If *n* is greater than 1, the sorption coefficient increases as the amount of compound sorbed on the solid phase increases; this indicates that the presence of sorbed compounds on the solid induces further sorption of additional compounds. If *n* is less than 1, sorption coefficient decreases when the amount of compound sorbed is increased; this indicates that the presence of sorbed compounds hinders further sorption [29].

### An alternative to K_d_: normalizing with organic carbon, K_oc_

Experimental determination of K_d_ values can be cost-prohibitive and time-consuming because one has to measure K_d_ at various conditions (e.g. different soil types, pH values, and organic and ionic strengths). When K_d_ is normalized to the organic carbon content of the soil, the organic carbon normalized sorption coefficient K_oc_ is obtained [1]:

$$K_{\mathrm{oc}}=\frac{K_{d}}{f_{\mathrm{oc}}}$$

However, mechanisms other than hydrophobic interactions are not accurately accounted for when normalization is performed using organic carbon content [1]. The differences between K_oc_ and K_d_ are observed in literature. Rabølle and Spliid [37] reported K_d_ and K_oc_ values ranging from 8.3-128 L/kg and 553-7988 L/kg, respectively, for tylosin in 4 different soils. Lertpaitoonpan et al. [33] reported K_d_ values for sulfamethazine for 5 different soils at varying pH between 0.23-3.91 L/kg, and K_oc_ values between 30.4-139.7 L/kg. In both cases, the antibiotics have higher K_oc_ values, which would suggest that the compounds are less mobile than their K_d_ values would indicate. Thus, while normalizing partition coefficients may help reduce variation between samples, it cannot be universally applied to all antimicrobials, particularly those that have ionizable functional groups.

### Case studies: sorption behavior of sulfonamides and macrolides in sediment

Macrolides and sulfonamides are commonly used antibiotic classes in livestock. Approximately 165800 kg of tylosin (a macrolide), 18660 kg of sulfamethazine, and 19400 kg of sulfathiazole are used annually in the United States for growth promotion, prevention, and therapy [42]. Our laboratory conducted sorption experiments for sulfamethazine and tylosin under varying pH, OM content, and ionic strengths using loam and sandy loam sediments. A study by Kim et al. [43] found sulfamethazine, erythromycin-hydrochloride, and tylosin in agricultural soils at concentrations of 9.1, 30.7, and 19.6 μg/kg, respectively. Therefore, sorption tests were performed using aqueous concentrations between 1-1000 μg/L prior to partitioning in order to mimic environmentally relevant concentrations of these antibiotics. Details regarding the methodology used to perform these batch experiments can be found in Additional file 1.

The pH-dependence of antibiotic sorption is critical, because many pharmaceuticals have acid-base properties resulting in changes in the overall net charge of the molecule as ammonia concentration in manure changes [6]. These factors can alter the distribution between the aqueous and solid phase, particularly for ionizable compounds [23]. Changes in soil pH can also affect surface charge and cation exchange capacity of the soil [1]. Ionic strength variations can lead to changes in pH, and cause electrostatic competition between ions present in the solution and the analyte of interest [6,44-46]. This study makes use of sediments that have similar OM content but have different fractions of sand, silt and clay. Most of the study conditions render a percentage of the compound in its ionized form, and due to the dependence on ionic strength, the antibiotics in the cationic form show increased sorption. However, sediment buffering capacity must be considered. The higher clay content in the loam sediment has a weaker buffering capacity relative to OM [47]. The sandy loam can more readily adjust the pH closer to the original pH, and therefore antibiotic sorption in sandy loam is less affected by changes in pH. The water solubility of the antibiotics increases with increase in dissolved OM content [48], which in turn results in increased mobility of antibiotics in soil [1]. Thus, it is important to understand how the K_d_ changes for each antibiotic when OM is present in the system.

### Sulfamethazine

Sulfonamides, or sulfa drugs, are synthetic antimicrobial agents containing the sulfonamide functional group (-RSO_2_NH_2_) [10]. Sulfonamides are mobile antibiotics and their speciation changes with pH. A common sulfonamide antibiotic is sulfamethazine (pk_a_: 1.62, 7.91), and its K_d_ values for various soil types reported in literature are presented in Table 1, and compared with the K_d_ values obtained experimentally from our laboratory.

### Effects of pH on sulfamethazine sorption

**Figure 2:**
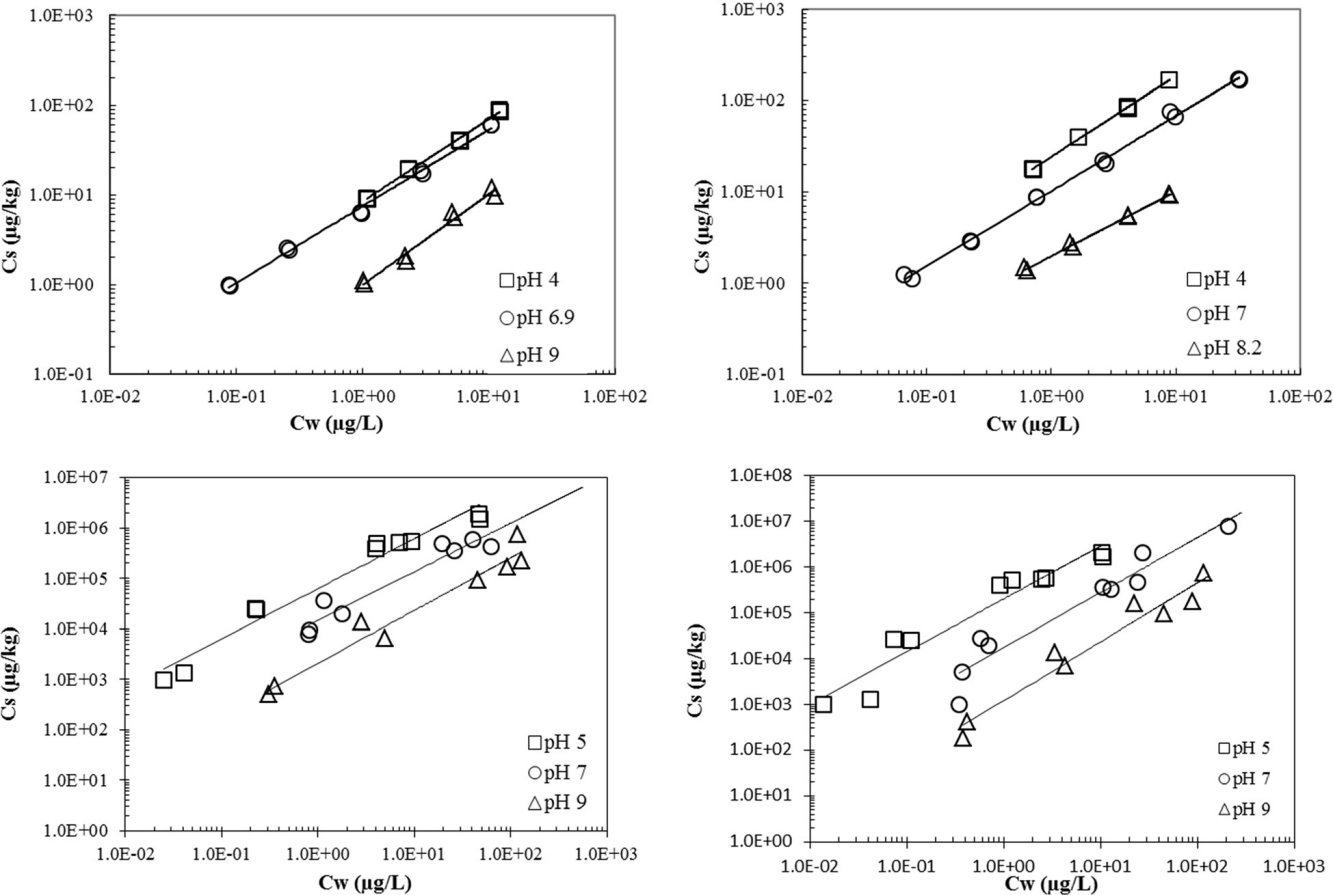
**Sulfamethazine sorption isotherms.** Top: Sulfamethazine sorption isotherms in sediment at low, neutral, and high aqueous pH. Left: sandy-loam and Right: loam Bottom: Tylosin sorption isotherms in sediment at low, neutral, and high aqueous pH. Left: sandy-loam and Right: loam

**Figure 3:**
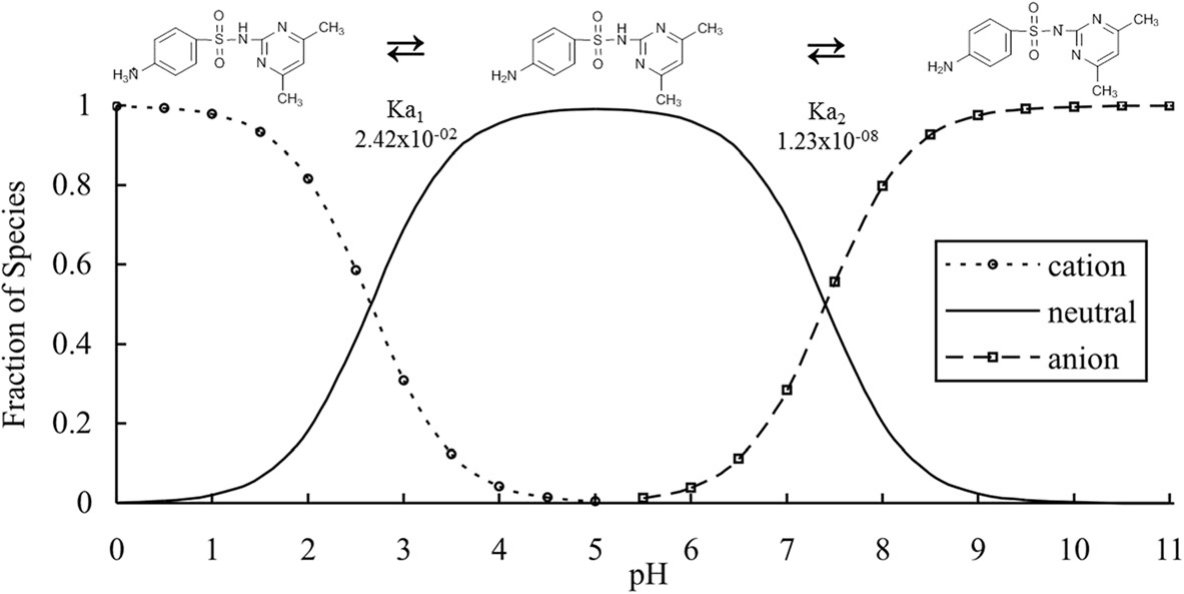
**Distribution of sulfamethazine species by pH.** Sulfamethazine is predominantly cationic below pH 1.62, neutral between pH 1.62 – 7.91, and anionic above pH 7.91. The chemical structures that represent the highest fraction of species is shown above the curve.

The sorption isotherm we determined for sulfamethazine (Figure 2) illustrates that K_d_ generally decreases with increase in pH for both loamy sand and loam sediments. This sorption behavior is consistent with the changes in the fraction of ionization of sulfamethazine as it converts from its cationic form to the neutral and anionic forms (See Figure 3). Positively charged species are electrostatically attracted to the negatively charged soil surface, and therefore a higher K_d_ is observed at pH below 5 (Table 3) [2,30]. Despite the presence of a small fraction of negatively charged sulfamethazine at pH 7, cation bridging does not appear to play a significant role in the sorption of sulfamethazine because sulfonamides interact primarily with soil organic matter via hydrophobic interactions [49]. This behavior of sulfonamides is in contrast with tetracycline and fluoroquinolone family of antibiotics that interact with soils primarily through cation exchange, surface complexation and cation bridging sorption mechanisms.

**Table 3:** Sulfamethazine and tylosin partitioning with changes in pH

		**Sandy loam**				**Loam**			
**Antibiotic**	**pH**	**K**_**d**_**(L/kg)**	**R**^**2**^	**K**_**f**_	**R**^**2**^	**K**_**d**_**(L/kg)**	**R**^**2**^	**K**_**f**_	**R**^**2**^
Sulfamethazine	5	6.9 ± 0.5	0.996	8.49	0.997	18 ± 1	0.998	24.3	0.999
	7	5.1 ± 0.3	0.997	7.54	0.996	5.3 ± 0.7	0.985	10.3	0.998
	9	0.9 ±0.3	0.954	0.994	0.980	0.9 ± 0.1	0.990	1.98	0.995
Tylosin	5	3x10^4^ ± 1x10^4^	0.933	6.28x10^4^	0.967	1.7x10^5^ ± 4x10^4^	0.952	2.02x10^5^	0.933
	7	1.5x10^4^ ± 6x10^3^	0.899	1.43x10^4^	0.950	2.2x10^4^ ± 7x10^3^	0.933	1.66x10^4^	0.867
	9	7x10^3^ ± 3x10^3^	0.872	2.21x10^3^	0.962	1.7x10^3^ ± 9x10^2^	0.808	1.26x10^3^	0.938

Note: Values were experimentally determined. Errors represent standard deviation. Methodology can be found in the additional files. Sulfamethazine data was performed using method 1 and tylosin data with method 2. Sulfamethazine batch experiments were performed with concentrations of 1, 3, 5, 10, 20, 30, 50, 100, and 300 ng/mL, and tylosin batch experiments were performed with concentrations of 1, 5, 10, 100, 200, 500, and ng/mL.

Sulfamethazine sorption (Table 3) trends towards linear isotherms in the sandy loam (n_pH5_ = 0.916, n_pH7_ = 0.853, and n_pH9_ = 1.01) at the three pH values tested. Sorption in loam exhibits some non-linearity (n_pH4_ = 0.885, n_pH6.9_ = 0.822, and n_pH8.2_ = 0.708). The n values are less than 1 which signifies that the sorption coefficient decreases when the amount of compound sorbed is increased, indicating that the presence of sorbed compounds hinders further sorption of antibiotic [29,50].

### Effects of ionic strength on sulfamethazine sorption

Sulfamethazine showed a slight decrease in sorption when ionic strength was increased from 50 mM to 250 mM (Table 4). The small decrease in sulfamethazine sorption may be attributed to a slight change in pH brought about by increase in ionic strength, and a possible change in interfacial potential between the negative sediment surface and the partially charged sulfamethazine [44-46]. The negatively charged soil surface reduces the sorption of anionic organic compounds [6,51].

**Table 4:** Sulfamethazine and tylosin partitioning with changes in ionic strength

		**Sandy loam**				**Loam**			
**Antibiotic**	**Ionic strength (mM)**	**K**_**d**_**(L/kg)**	**R**^**2**^	**K**_**f**_	**R**^**2**^	**K**_**d**_**(L/kg)**	**R**^**2**^	**K**_**f**_	**R**^**2**^
Sulfamethazine	30	10.3 ± 0.5	0.999	26	0.992	5.5 ± 0.8	0.991	46	0.978
	50	10 ± 1	0.994	47	0.991	7.5 ± 0.5	0.997	59	0.960
	250	7 ± 3	0.913	54	0.974	2.8 ± 0.3	0.995	84	0.917
Tylosin	30	500 ± 45	0.969	1640	0.969	190 ± 30	0.984	2689	0.919
	50	2.9x10^3^ ± 1x10^2^	0.999	4411	0.980	600 ± 300	0.886	3109	0.972
	250	1.4x10^4^ ± 7x10^3^	0.927	17200	0.935	9.6x10^3^ ± 7x10^2^	0.998	5555	0.937

Note: Values were experimentally determined. Errors represent standard deviation. Methodology can be found in the additional files. Sulfamethazine data was performed using method 1 and tylosin data with method 2. Sulfamethazine batch experiments were performed with concentrations of 17.7, 35.4, 53.1, 70.8, 88.5 ng/mL, and tylosin batch experiments were performed with concentrations of 10, 100, 500, and 1000 ng/mL.

### Effects of organic matter on sulfamethazine sorption

Fan et al. [31] found that the sorption correlation of sulfamethazine with OM is confounded by soil pH. Thiele-Bruhn and Aust [52] observed that when electrostatic competition were eliminated through use of an acidic pig slurry matrix, the sorption of sulfonamides decreased. Decreased antibiotic sorption may be attributed to association of sediments with OM from manure components (ammonia–N-containing soluble hydrocarbons such as amino acids urea [53,54], and N-heterocyclic hydrocarbons such as pyrrols, methylindols, and nitrogen bases [55]). The interaction of OM with soil can block access of antibiotics to interlayer sorption sites in soil [25,52,56]. In our study, we found no consistent trend with changes in humic acid (HA) concentrations (Table 5). These tests may have been complicated by the presence of both dissolved and suspended HA in solution. Suspended HA provides sites where additional partitioning can occur. Increased amounts of dissolved OM can cause antibiotics to desorb from soil, and increased association of antibiotics with dissolved OM can facilitate transport in the environment [1,16,57]. Furthermore, any anionic sulfamethazine may be repelled by the increased surface charge occurring from the dissolved OM [30].

**Table 5:** Sulfamethazine and tylosin partitioning with changes in organic strength

		**Sandy loam**				**Loam**			
**Antibiotic**	**Humic acid**	**K**_**d**_	**R**^**2**^	**K**_**f**_	**R**^**2**^	**K**_**d**_	**R**^**2**^	**K**_**f**_	**R**^**2**^
	**(mg/L)**	**(L/kg)**				**(L/kg)**			
Sulfamethazine	1	9 ± 2	0.974	31	0.910	5 ± 2	0.918	70	0.913
	10	3 ± 2	0.727	67	0.947	4 ± 2	0.878	84	0.827
	50	12 ± 3	0.960	50	0.910	6 ± 2	0.961	86	0.958
Tylosin	1	60 ± 30	0.869	1.43x10^5^	0.873	50 ± 10	0.960	1.66x10^5^	0.983
	10	90 ± 20	0.976	4.41x10^3^	0.940	50 ± 50	0.715	1.77x10^4^	0.829
	50	140 ± 70	0.936	8.18x10^4^	0.996	40 ± 30	0.714	1.19x10^5^	0.975

Note: Values were experimentally determined. Errors represent standard deviation. Methodology can be found in the additional files. Sulfamethazine data was performed using method 1 and tylosin data with method 2. Sulfamethazine batch experiments were performed with concentrations of 17.7, 35.4, 53.1, 70.8, 88.5 ng/mL, and tylosin batch experiments were performed with concentrations of 10, 15, 30, and 50 mg/L.

### Tylosin

Macrolides, which are mainly active to Gram-positive bacteria, inhibit ribosomal protein synthesis. Their activity stems from the presence of the macrolide ring, a large lactone ring to which one or more deoxy sugars are attached [58]. A case study on the soil sorption of tylosin antibiotic, which belongs to the macrolide class, is presented below.

### Effects of pH on tylosin sorption

**Scheme 1:**
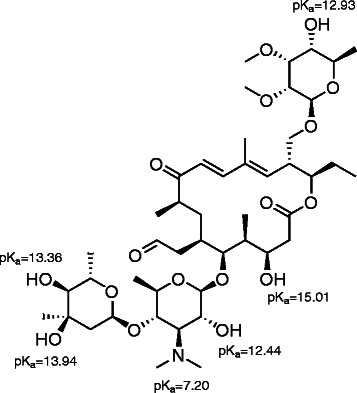
**The macrolide, tylosin.** Chemical structure and pK_a_ values are shown.

Tylosin sorption (pK_a_: 7.20, 12.44, 12.93, 13.36, 13.94, and 15.01; assignments of pKa values in the molecule are shown in Scheme 1) strongly depends on the pH, as well as on the surface area, clay content, and cation-exchange capacity of the soil [2]. Since tylosin is water soluble (5 mg/mL) and has high molecular weight, it is unlikely that sorption occurs through penetration of soil micro pores [25]. Several studies have reported that the K_d_ values for tylosin increase with decreasing pH [6,25,38,59]. The same pH effects on the sorption behavior of tylosin were observed in the studies conducted in our laboratory, as shown in Figure 2 and in Table 3. Tylosin sorption increased in both loam and sandy loam sediments when the pH of the sediment-aqueous system was decreased. The increased sorption of tylosin at pH 5, relative to its sorption at pH 7 and 9 can be expected due to the shift in tylosin speciation towards the positively charged species, resulting in increased electrostatic attractions to the negatively charged sediment surface [25].

Our experimental values indicate that tylosin sorption (Table 3) is linear over 3 orders of magnitude in the sandy loam sediment at pH 5 and 7 (n_pH5_ = 0.993, n_pH7_ = 1.05). However, at pH 9, n_pH9_ = 1.22; this greater than unity value indicates a non-linear sorption behavior that can be attributed to the presence of sorbate molecules inducing further sorption [29]. In other words, the presence of the sorbed tylosin results in further sorption of the antibiotic in soil. Similarly, the loam sediment exhibits some non-linearity (n_pH5_ = 1.15, n_pH7_ = 1.18, n_pH9_ = 1.19), but to a lower extent. Thus, electrostatic forces dominate the sorption model.

### Effects of ionic strength on tylosin sorption

Literature suggests that tylosin sorption decreases with increase in ionic strength due to the consequent change in pH, and as a result of competition between the electrolyte cations and the positively charged tylosin species for negatively charged sorbent [6]. However, ionic strength experiments performed in our laboratory at a constant pH of 7, with tylosin in mostly neutral form, showed a reverse trend (Table 4). Instead, at pH 7, tylosin sorption increased with increase in ionic strength. This sorption behavior may be attributed to the presence of hydrated cations in the solution (Ca^2+^, Na^+^) that may act as proton-donors, which can protonate the tertiary amine in the tylosin molecule and enhance its sorption properties at higher ionic strengths. Yong-Hak et al. [60], observed that the tertiary amine group of erythromycin can become protonated, and that clay surfaces can facilitate this with their proton supplying power. Alternatively, hydrated cations that adsorb on the negatively charged soil can provide hydrogen bonding as an important sorption mechanism for tylosin because of several OH groups present in the molecule.

### Effect of organic matter on tylosin sorption

It was expected that the K_d_ values for tylosin would decrease in the presence of OM due to increased solubility. However, studies in our laboratory demonstrated higher K_d_ values with increased OM (represented as humic acids) using 10 ppm tylosin in sandy loam, and unchanged values in loam (Table 5). Similar to sulfamethazine, complications could arise from the presence of both dissolved and suspended OM within the solution. This complexity can be observed in the change in K_f_ values with increasing tylosin concentration (Table 5). Likewise, differences in the sorbates can also influence sorption, as was observed in the sorption of tylosin to the two sediment types used in our laboratory study.

### Sorption of tylosin metabolites

**Figure 4:**
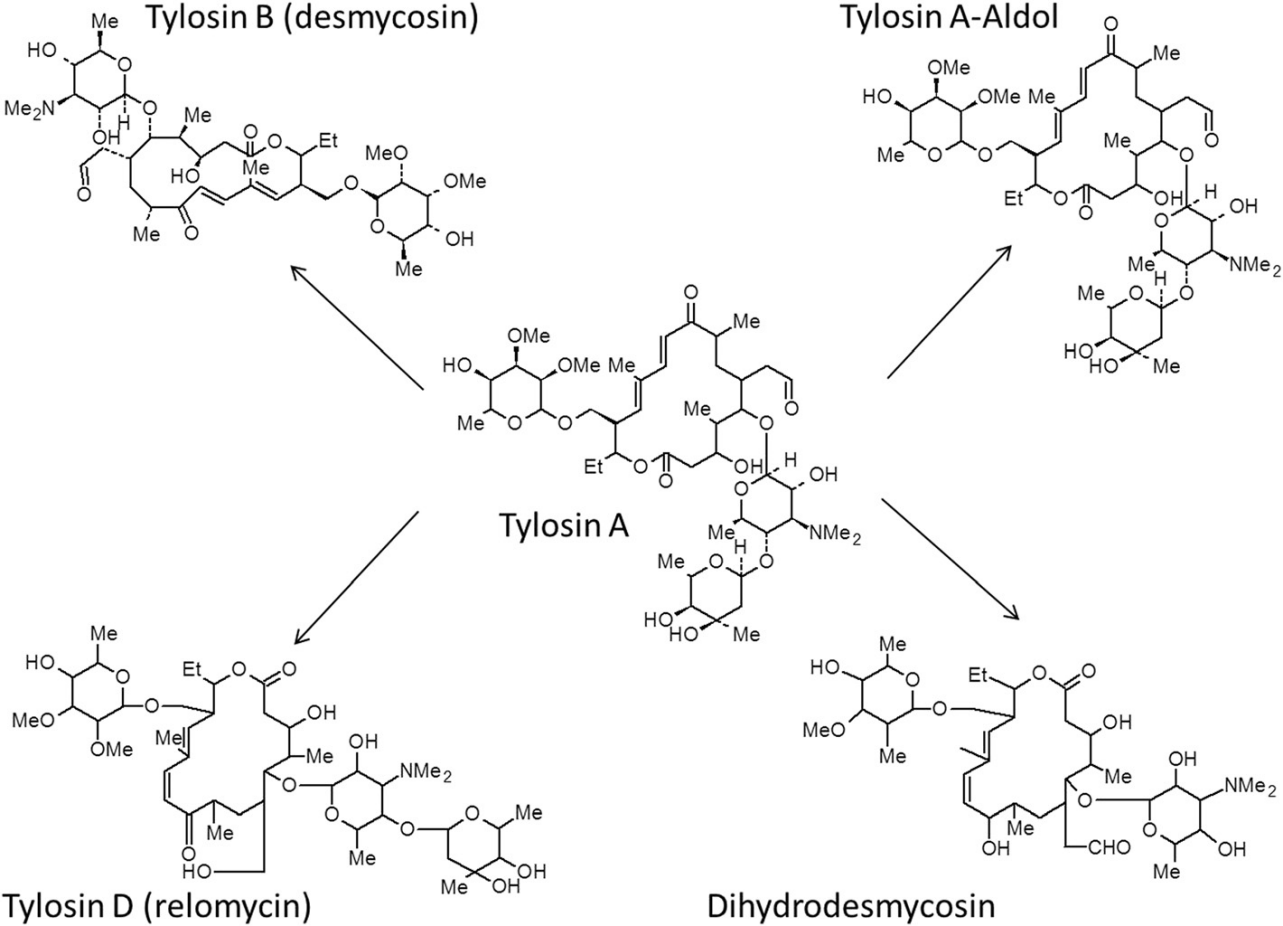
**Degradation products of tylosin.** Under environmental conditions, tylosin A can degrade to desmycosin, relomycin, dihydrodesmycosin, and tylosin A-Aldol. Tylosin A, relomycin, dihydrodesmycosin,desmycosin, and additional unknown degradates are present in swine excreta [62,63].

Tylosin and other antibiotics may interconvert between multiple chemical forms depending on environmental conditions as shown in Figure 4. Tylosin A and its related compounds are stable from pH 4-9 [61]. Metabolism of tylosin by livestock results in the excretion of tylosin A, B, D, and dihydrodesmycosin metabolites [2,62,63]. As the metabolites retain different degrees of bioactivity (TA = 100%, relative, TB = 83%, TD = 35%, dihydrodesmycosin = 31%) [2], it is important to consider the speciation of tylosin present in the environment. Tylosin A, D, and tylosin A-Aldol have been found to exhibit similar sorption characteristics [2]. However differences in sorption behavior between tylosin A (K_ow_: 0.552-32.659) and its hydrolysis product tylosin B (spiramyycin, K_ow_: 1.535-78.343) may be significant. Tylosin B results from the hydrolysis of tylosin A which involves a loss of the mycarose ring attached at position 4 of the 16-membered lactone ring. With this loss, the hydrophilicity of tylosin increases. This can alter tylosin’s potential to sorb to soil through hydrophobic interactions, and tylosin B can potentially be more mobile in the environment. Therefore, due to the varying properties of the different forms of tylosin, including tylosin A, B, C, and D it may not be appropriate to use only one K_d_ value for risk assessment of tylosin. Rather, K_d_ values should be obtained for all forms possible under the expected conditions.

To date, studies on the environmental fate of tylosin A degradation products are very limited [2,24]. Our laboratory determined sorption differences between tylosin A and B in loam sediment at an initial concentration of 50 μg/mL equilibrated between sediment and aqueous phase for 24 h. The amount of tylosin remaining in the aqueous phase was determined by liquid chromatography coupled to an ion trap mass spectrometer (LC-MS) following concentration by solid phase extraction (SPE). The methodology used follows that in method 2 of the additional file 1. It was found that 53% tylosin A and 39% tylosin B were sorbed in the loam. However, these results may be complicated by the hydrolysis of tylosin over the equilibration time period and during the SPE process. A study by Ali et al. [64] observed a decrease in sorption with decreasing pH. This result is contradictory to what is found in most of the studies reported in the literature. The decrease may be associated with the decrease in tylosin A due to the formation of tylosin metabolites. The conversion of the parent compound to metabolites and the interconversions that occur under varying conditions are challenges associated with analyzing degradation products.

## Conclusions

It is not possible to determine the fate and mobility of antibiotics and antimicrobials in the environment with K_ow_ values alone. The variability in mobility, as demonstrated by K_d_ and K_f_ values due to environmental factors such as pH, ionic strength, and organic strength as well as the multiple chemical functions of the molecule are large. In the case studies presented here, sulfamethazine was found to be very mobile in sandy loam and loam sediments, while tylosin is very immobile in both sediments. It is possible that degradates may have a higher mobility than the parent compounds. It is also important to determine sorption coefficients of antibiotics at environmentally-relevant concentrations. To achieve this, highly sensitive analytical techniques must be used, including the use of radiolabeled compounds.

A decrease in solution pH resulted in an increase in sorption of the cationic forms of antibiotics suggesting that electrostatic forces are the favored sorption mechanism of sulfamethazine and tylosin. As with other known pharmaceuticals, ionization of these compounds at the conditions considered was shown to favor the sorption of compounds. A cation-exchange mechanism can also be envisioned based on the results of ionic strength experiments where ions compete with charged species for sites on the soil. Organic matter dependence of K_d_ appears to be concentration-dependent, where low antibiotic concentrations result in higher soil sorption, and higher antibiotic concentrations result in lower soil sorption. It is observed that sorption mechanisms are much more complex than simple hydrophobicity and hydrogen bonding, and should also consider van der Waals and electrostatic interactions, as well as cation-exchange, competition and bridging. Additionally, the properties of the sorbent also affect the sorption process. Differences in clay content alone provide notable changes in K_d_ values. Finally, sorption of antibiotics in soil, manure, and biosolids can be microbially-mediated, and may result in degradation or possibly irreversible binding onto manure solids with time [25,40,62,65]. Fate and transport studies should take into account not only K_d_ values for the parent compounds, but also those of the transformation products formed during biotic and abiotic processes in soil.

## Abbreviations

VPs: Veterinary pharmaceuticals; Kow: Octanol-water partition coefficient; Dow: pH dependent octanol-water distribution ratio; Kd: Soil-water partition coefficient; Cs: Concentration of compound in soil; Cw: Concentration of dissolved compound in water; PAH: Poly aromatic hydrocarbon; Koc: Organic carbon normalized sorption coefficient; OM: Organic matter; DOM: Dissolved organic matter; HA: Humic acid; TA: Tylosin A; TB: Tylosin B; TD: Tylosin D; LC-MS/MS: Liquid chromatography tandem mass spectrometry; SPE: Solid phase extraction.

## Competing interests

The authors declare that they have no competing interests.

## Authors’ contributions

SRW, DAGN, and LZ performed experiments and analyzed data. SRW drafted the manuscript. DSA devised the study, secured funding, and supervised personnel during sampling, analysis and manuscript preparation. All authors read and approved the final manuscript.

## Supplementary Material

Additional file 1: Methodology for Sorption Batch Experiments. Click here for file
