# Supplementary material for: Assessing antibiotic sorption in soil: a literature review and new case studies on sulfonamides and macrolides
Source: Chem Cent J. 2014 Jan 17;8:5. doi: 10.1186/1752-153X-8-5 (PMC3905979; doi:10.1186/1752-153X-8-5)
Supplement: Additional file 1: — Methodology for Sorption Batch Experiments. [file 1752-153X-8-5-S1.pdf]

Additional File

Methodology for Sorption Batch Experiments

Sorption isotherms for the veterinary antibiotics erythromycin (ERY), sulfamethazine (SUL), and tylosin (TYL) were determined using the batch equilibrium method. Briefly, a predetermined amount of soil and either 10 mM $CaCl_2$ solution or Nanopure™ water were placed in a sterile polypropylene or glass centrifuge tubes. Duplicate samples at each concentration level were used to establish antibiotic sorption isotherms for each of the two soil types (loam and sandy loam). The test mixtures in centrifuge tubes were equilibrated at room temperature using an end-over-end mechanical shaker, and centrifuged at 1500 rpm. For samples that required larger volumes, equilibration was performed in glass bottles using a plate shaker; and the solid portion was allowed to settle for 12 h. Aliquots of the supernatant were collected for either radioactivity measurement (**Method 1**) or LC-MS analysis (**Method 2, Method 3**). The soil:solution ratios, equilibration time and working concentrations were all optimized for all analytes prior to the actual batch experiments. Soil:solution ratios are ideal when greater than 20% and less than or equal to 50% of the analyte is sorbed [1].

Effects of pH, ionic strength and humic acid (HA) concentrations were also investigated by changing the conditions for the aqueous phase used. Solution pH was adjusted using HCl or NaOH to 3, 5, 7, and 9 while maintaining the ionic strength at 30 mM. Ionic strengths of 50 mM and 250 mM were prepared by adjusting the ionic strength of the 0.01M $CaCl_2$ solution using NaCl. The amount of NaCl used was based on the equation: ionic strength = $\sum (\mu_i^2 c_i)$ where μ_i = charge of the cation or the anion being considered, and c = concentration of the cation or anion in solution in moles per liter.

To study the influence of HA concentrations on the sorption of ERY, SUL, and TYL, the HA levels were varied by spiking the soil with 1, 10 and 50 ppm HA prepared in 0.01 M $CaCl_2$, while maintaining the pH at 7 and ionic strength at 30mM. Control and blank set-ups were prepared for all tests. Control set-ups contained the analyte solution in 0.01M $CaCl_2$ only (no soil), while the blank set-up contained soil suspended in 0.01M $CaCl_2$ without any spiked analyte. The control set-ups were used to check for container sorption. All results were corrected accordingly. All set-ups were performed in duplicates.

Method 1: Liquid Scintillation Counting for the Quantification of ^{3}H-Erythromycin and ^{14}C-Sulfamethazine

For erythromycin (ERY-^{3}H, 80 Ci/mmol activity purchased from American Radiolabeled Chemicals, Inc, St. Louis, Mo.) and sulfamethazine (SUL-^{14}C, 8.2 mCi/mmol activity was generously donated by Dr. Joel Coats from Iowa State University), the availability of radiolabeled compounds allowed the detection of lower sorbate concentrations, at environmentally relevant concentrations, using the TriCarb 1600 TR liquid scintillation counter (Packard, Ramsey, MN).

The ERY-^{3}H set ups were spiked with radiolabeled material equivalent to 7.34, 8.32, 9.25, 18.5, and 27.7 pg/mL concentrations for soil:solution ratio of 100 mg:5 mL, followed by a 24 h equilibration time. The SUL-^{14}C set-ups were spiked with radiolabeled material equivalent to 17.7, 35.4, 53.1, 70.8, 88.5 ng/mL concentrations for soil:solution ratio of 500 mg:5 mL (1:10 soil:solution ratio) followed by a 98-h equilibration time. A second experiment with SUL-^{14}C was performed using the same experimental setup with the exception of a wider concentration range that include 1, 3, 10, 20, 30, 50, 100, and 300 ng/mL. The solid and liquid portions were

separated by centrifugation at 1500 rpm. One mL aliquots were taken from the liquid phase, transferred to a 5-mL scintillation vial to which 3-mL scintillant were added for measurement of the radioactivity remaining in solution. The resulting solutions were measured against 0.01M $CaCl_2$ background. Samples were quantified using 5 standards: 4.62, 6.93, 9.25, 23.1, and 46.2 pg/mL for ERY-^{3}H, and 10.6, 17.7, 35.4, 70.8, and 106 ng/mL for SUL-^{14}C. The radioactivity of the sorbed compound in soil was calculated by subtraction, based on the original radioactivity spiked into the system.

Method 2: Quantification of Tylosin by Liquid Chromatography Coupled to an Ion Trap Mass Spectrometer

Because no commercial radiolabelled TYL was available, non-radiolabelled TYL (tylosin tartate was purchased from Sigma Chemical Co., St. Louis, MO.) was used at 10, 100, 500, and 1000 ng/mL spiking concentrations. The use of higher concentrations of TYL was necessary because of the detection limit on the HPLC-MS instrument. The soil:solution ratio of 1 g:40 mL 0.01M $CaCl_2$ solution was determined to fall within 20-80% sorption of TYL in sediment based on preliminary experiments. A 24-h equilibration time was determined to be sufficient to reach equilibrium. Aliquots from the supernatant were then pre-concentrated by solid phase extraction (SPE), using OASIS™ HLB cartridges from Waters (Milford, MA). Samples for SPE were first treated with 20mL 0.05M citric acid buffer (pH 3.8), 20 mL 0.01M Na_2EDTA then diluted to 250 mL (final solution pH ~4). SPE cartridges were conditioned using the following scheme: 3mL ACN, 3mL ethyl acetate (EtOAc), 3mL ACN, and 3 mL H_2O. The samples were then loaded onto conditioned cartridges at an average flow rate of about 7mL/min (equivalent to SPE box pressure of ~8 torr). After 5 minutes of drying, the cartridges were eluted using 3 mL

acetonitrile followed by 3 mL ethyl acetate. The percent recovery of TYL using these conditions was 107 ± 5%. Eluates were dried under a slow stream of air, and reconstituted in 1000 uL 10% methanol/90% water. All samples were spiked with 100 ng/mL roxithromycin as an internal standard prior to injection.

Liquid chromatography mass spectrometry (Thermo Fisher Scientific Surveyor coupled to Thermo Fisher Scientific LCQ Advantage, San Jose, CA) was employed for the analysis of TYL from the batch equilibrium experiments. To separate TYL-A from its known hydrolysis forms, TYL-B (*Desmycosin)*, TYL-C (*Macrosin)* and TYL-D *(Relomycin)*, a mobile phase with a 15-min gradient elution was applied. Separation was performed on a C-18 column (Thermo BetaBasic 100 x 2.1mm C-18 column with 3-μm particle diameter) using 20:80 acetonitrile:water (with 0.1% formic acid). The flow rate of the mobile phase was 0.300 mL/min. From the initial conditions, the percentage of acetonitrile was increased to 90% from 0-4 min, and was held constant 4 min. Finally, the mobile phase was brought back to initial conditions in 2 min, which was then maintained for 5 minutes. Detection of TYL was achieved using electrospray ionization in positive mode. Mass spectrometric acquisition was performed by single ion monitoring MS parameters for this method are listed in **Table A1**. Samples were quantified based on an external calibration curve constructed using 10, 40, 100, 400 and 1000 ng/mL TYL, normalized by the signal of an internal standard containing 100 ppb roxithromycin.

Method 3: Quantification of Tylosin by Liquid Chromatography Tandem Mass Spectrometry

A second set of sorption experiments for TYL were performed using a wider concentration range that includes 1, 5, 10, 100, 200, 500, and 1000 ng/mL spiking

concentrations. The soil:solution ratio of 0.5 g:400 mL Nanopure™ water (1:1000) was determined in a preliminary study, at which approximately 40-50% of the TYL will sorb in sediment. A 24-h equilibration time was also determined to be sufficient to reach equilibrium. Aliquots from the supernatant were collected and filtered with 0.45 μm polypropylene membrane syringe filters (VWR) for direct analysis (without SPE) using a more sensitive HPLC-MS system (LC-MS/MS, Agilent 1100 Series coupled to Agilent 6410, Sta. Clara, CA). Isocratic separation in HPLC was performed using 60/40 acetonitrile/water (with 0.3% formic acid) on a C-18 column (Thermo BetaBasic 100 x 2.1mm C-18 column with 3-μm particle diameter) at 0.200 mL/min. An internal standard of roxithromycin was added for a final concentration of 25 ng/mL. Detection of TYL was achieved using electrospray ionization in positive mode. Mass spectrometric acquisition was performed by single reaction monitoring. Tylosin, parent *m/z* of 916.5, was monitored with a quantifying ion of 174.1 and a qualifying ion of 772.1. Roxithromycin, parent *m/z* of 837.1, was monitored with a quantifying ion of 158.1 and a qualifying ion of 679.4. The MS parameters for this method are listed in **Table A2**. Samples were quantified based on single-point standard addition (by adding TYL to reach a final concentration of 25 ng/mL standard added to each sample) to reduce matrix effects associated with calibration by an external curve.

Table A1. Method 2 Mass Spectrometry Parameters.

Dry gas flow rate (L/min)	10
Nebulizer flow (psi)	35
Dry temperature (^{0}C)	350
Capillary voltage (V)	4000
Fragmentor voltage (V)	190
Mass fragments	916 $[M+H]^+$ Tylosin A<br>772 $[M+H]^+$ Tylosin B<br>902 $[M+H]^+$ Tylosin C<br>837.5 $[M+H]^+$ Roxithromycin

Table A2. Method 3 Mass Spectrometry Parameters.

Dry gas flow rate (L/min)	11
Nebulizer flow (psi)	35
Dry temperature (^{0}C)	350
Capillary voltage (V)	4000
Fragmentor voltage (V)	270
Mass transitions	916.5 → 174.1, 772.1 (Tylosin A)<br>837.1 → 158.1, 679.4 (Roxithromycin)

References

1. OECD/OCDE: **OECD Guideline for the Testing of Chemicals: Adsorption-Desorptioin Using a Batch Equilibrium Experiment.** In *Book OECD Guideline for the Testing of Chemicals: Adsorption-Desorptioin Using a Batch Equilibrium Experiment* (Editor ed.^eds.). City: OECD Publishing; 2000.
